# Characterisation of the *mgo *operon in *Pseudomonas syringae *pv. *syringae *UMAF0158 that is required for mangotoxin production

**DOI:** 10.1186/1471-2180-12-10

**Published:** 2012-01-17

**Authors:** Eva Arrebola, Víctor J Carrión, Francisco M Cazorla, Alejandro Pérez-García, Jesús Murillo, Antonio de Vicente

**Affiliations:** 1 Instituto de Hortofruticultura Subtropical y Mediterránea "La Mayora" (IHSM-UMA-CSIC), Estación Experimental La Mayora, Algarrobo-Costa, 29750 Málaga, Spain; 2 Instituto de Hortofruticultura Subtropical y Mediterránea "La Mayora" (IHSM-UMA-CSIC). Departamento de Microbiología, Facultad de Ciencias, Universidad de Málaga, Unidad Asociada al CSIC, Campus de Teatinos, 29071 Málaga, Spain; 3 Laboratorio de Patología Vegetal, ETS de Ingenieros Agrónomos, Universidad Pública de Navarra, 31006 Pamplona, Spain

## Abstract

**Background:**

Mangotoxin is an antimetabolite toxin that is produced by strains of *Pseudomonas syringae *pv. *syringae*; mangotoxin-producing strains are primarily isolated from mango tissues with symptoms of bacterial apical necrosis. The toxin is an oligopeptide that inhibits ornithine N-acetyl transferase (OAT), a key enzyme in the biosynthetic pathway of the essential amino acids ornithine and arginine. The involvement of a putative nonribosomal peptide synthetase gene (*mgo*A) in mangotoxin production and virulence has been reported.

**Results:**

In the present study, we performed a RT-PCR analysis, insertional inactivation mutagenesis, a promoter expression analysis and terminator localisation to study the gene cluster containing the *mgo*A gene. Additionally, we evaluated the importance of *mgo*C, *mgo*A and *mgo*D in mangotoxin production. A sequence analysis revealed an operon-like organisation. A promoter sequence was located upstream of the *mgo*B gene and was found to drive *lac*Z transcription. Two terminators were located downstream of the *mgo*D gene. RT-PCR experiments indicated that the four genes (*mgo*BCAD) constitute a transcriptional unit. This operon is similar in genetic organisation to those in the three other *P. syringae *pathovars for which complete genomes are available (*P. syringae *pv. *syringae *B728a, *P. syringae *pv. *tomato *DC3000 and *P. syringae *pv. *phaseolicola *1448A). Interestingly, none of these three reference strains is capable of producing mangotoxin. Additionally, extract complementation resulted in a recovery of mangotoxin production when the defective mutant was complemented with wild-type extracts.

**Conclusions:**

The results of this study confirm that *mgo*B, *mgo*C, *mgo*A and *mgo*D function as a transcriptional unit and operon. While this operon is composed of four genes, only the last three are directly involved in mangotoxin production.

## Background

Antimetabolite toxins are generally small metabolites that exhibit strong effects in plant cells by causing an increase in disease symptoms [1]. Various toxic substances produced by pathovars of *Pseudomonas syringae *have been well characterised. Each antimetabolite toxin inhibits a specific step in the glutamine and arginine biosynthesis pathways of the host, enhancing disease symptoms and increasing the virulence of the bacterial pathogen. The most well-studied antimetabolite toxins are tabtoxin and phaseolotoxin [2].

Tabtoxin is a monocyclic β-lactam that specifically inhibits the enzyme glutamine synthetase (GS, EC 6.3.1.2). This toxin is produced by *P. syringae *pv. *tabaci*, pv. *coronafaciens *and pv. *garcae *[3]. The biosynthetic pathway of tabtoxin is not well understood, and tabtoxin biosynthesis may diverge from the lysine biosynthetic pathway prior to the formation of diaminopimelate [4,5]. A genetic analysis of tabtoxin production revealed the presence of biosynthetic genes at the *att *site adjacent to the *lys*C tRNA gene in *Pseudomonas syringae *BR2 [6]. The various ORFs within this region include sequences similar to β-lactam synthase, clavaminic acid synthase and enzymes involved in amino acid synthesis. Additionally, novel ORFs were identified in a portion of the biosynthetic region that is known to be associated with a toxin hypersensitivity phenotype [6].

Phaseolotoxin is produced mainly by *P. syringae *pv. *phaseolicola *and pv. *actinidae*. The molecular structure of phaseolotoxin includes a sulphodiaminophosphinyl moiety linked to a tripeptide of ornithine, alanine and homoarginine [2]. Phaseolotoxin inhibits ornithine carbamoyltransferase (OCT, EC 2.1.3.3) [7]. The phaseolotoxin homoarginine and ornithine residues are synthesised by a transamidation reaction that requires arginine and lysine [8,9].

Aguilera *et al*. [10] have reported a biosynthetic cluster, *pht*, which is composed of 23 genes flanked by insertion sequences and transposases, that is involved in the biosynthesis of phaseolotoxin. Mutations of 11 of the genes within the cluster led to a Tox^- ^phenotype, and the mutation of three additional genes resulted in low levels of toxin production. Preliminary results also indicated that the product of *pht*L may be involved in the regulation of phaseolotoxin biosynthesis [10].

**Figure 1:**
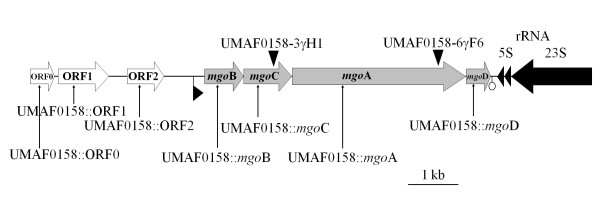
**Organisation of the DNA cloned into pCG2-6 and the locations of the insertional and mini*Tn5* mutants used in this study**. pCG2-6 contains an 11,103-bp insert of chromosomal DNA derived from *Pseudomonas syringae *pv. *syringae *UMAF0158 (GenBank accession number DQ532441). The site of insertion or mini*Tn5 *within the UMAF0158-3γH1 and UMAF0158-6γF6 mutants (▼) [15] as well as the design of the insertional mutants (↑) generated in the current study are indicated. The predicted sites of the putative promoters (►) and transcriptional terminators (○) are indicated. The putative *mgo *operon involved in mangotoxin production by UMAF0158 is illustrated by grey arrows. Each gene studied in this study was given a specific name. The ORFs upstream of the *mgo *operon are illustrated by white arrows, and the 5S and 23S ribosomal RNAs are indicated by black arrows.

*Pseudomonas syringae *pv. *syringae *(Pss) is a pathogenic bacterium that can cause canker, blossom blights and leaf spots in more than 200 different plant species, many of which are of economic importance [11]. Strains of this pathovar can cause bacterial apical necrosis on mango trees, limiting mango production in the Mediterranean area [12]. More than 86% of the Pss strains isolated from mango tissues produce mangotoxin, an antimetabolite toxin that inhibits ornithine N-acetyl-transferase (OAT), a key enzyme in the biosynthesis of arginine [13]. Mangotoxin also acts as a virulence factor that increases the necrotic symptoms of Pss strains during the infection of plant tissues [14]. In a previous study, a DNA fragment from Pss, UMAF0158, was cloned into pCG2-6 and sequenced (DQ532441), revealing a cluster of 4 ORFs that included the *mgo*A gene. Our group identified *mgo*A as the first *P. syringae *pv. *syringae *gene known to be directly involved in mangotoxin production [15]. This gene encodes a putative nonribosomal peptide synthetase (NRPS), and its inactivation by insertional mutagenesis abolishes mangotoxin production and drastically reduces virulence [14,15]. The genetic organisation of the three remaining genes and their roles in the production of mangotoxin remain unknown. The goal of our current study is to determine the organisation of the four ORFs in this cluster (Figure 1) and their relative importance in the production of mangotoxin.

## Results

The gene cluster containing *mgo*A may constitute an operon composed of four ORFs. Our current study provides insight into the organisation of the operon and the involvement of the genes in the production of mangotoxin.

### The construction and characterisation of insertion mutants derived from *Pseudomonas syringae *pv. *syringae *UMAF0158

Each ORF that was cloned into plasmid pCG2-6 (Figure 1) was subjected to insertional inactivation mutagenesis in the *P. syringae *pv. *syringae *UMAF0158 chromosome by integration of the appropriately cloned PCR products. The ORFs were 92%-98% identical to the homologous genes in *P. syringae *pv. *syringae *strain B728a (accession no. CP000075, Table 1). The deduced ORF0 and ORF1 protein products are homologous to proteins of the HAD hydrolase family and aldo-keto oxidoreductases, respectively. The mutation of these ORFs by insertional inactivation did not affect mangotoxin production. ORF2 is located just upstream of the putative *mgo *operon (Figure 1) and contains a putative ribosomal binding site (RBS) at nucleotide -6 (AAGAAGT). This gene is 97% identical to Psyr_5008 from *P. syringae *pv. *syringae *B728a (Table 1), PSPTO_5454 from *P. syringae *pv. *tomato *DC3000 and PSPPH_5087 from *P. syringae *pv. *phaseolicola *1448A. The protein products of the genes from each of these bacteria were annotated in the database as members of the GntR family of transcriptional regulators [16]. When ORF2 was disrupted, the corresponding mutant UMAF0158::ORF2 still produced mangotoxin (Tables 1 and 2).

**Table 1:** Characterization of disrupted genes surrounding the *mgo *operon in derivates mini*Tn5 *and insertional mutants from the wild type *Pseudomonas syringae *pv.*syringae *UMAF0158 mangotoxin producer

Bacterial strains	ORF disrupted	Mangotoxin production^a^	Putative homology of disrupted gene	Comparison ncl-ncl^b ^with Pss B728a	
				
				% of identity	gene name
*miniTn5 mutants*^c^					
UMAF0158-3νH1	*mgo*C	-	Conserved hypothetical protein	95	Psyr_5010
UMAF0158-6νF6	*mgo*A	-	Nonribosomal peptide synthetase	93	Psyr_5011
*Insertional mutants*					
UMAF0158::ORF0	ORF0	+	HAD hydrolase	92	Psyr_5006
UMAF0158::ORF1	ORF1	+	Aldo-keto oxidoreductase	98	Psyr_5007
UMAF0158::ORF2	ORF2	+	Transcriptional regulator GntR family	97	Psyr_5008
UMAF0158::*mgo*B	*mgo*B	(+)	Haem-oxigenase-like^e^	96	Psyr_5009
UMAF0158::*mgo*C	*mgo*C	-	p-aminobenzoate N-oxygenase AurF^e^	95	Psyr_5010
UMAF0158::*mgo*A	*mgo*A	-	Nonribosomal peptide synthetase	93	Psyr_5011
UMAF0158::*mgo*D	*mgo*D	-	Poliketide_cyc2^d^	94	Psyr_5012

a) Presence of inhibition halo around the bacterial growth point in *E. coli *growth inhibition test. -: absence of inhibition halo, +: presence of inhibition halo, (+): slight mangotoxin production (smaller and opaque halo)

b) ncl-ncl is used as abbreviation of nucleotide-nucleotide

c) mini*Tn5 *mutants defective in mangotoxin production obtained in a previous work [15]

d) Putative function by family domains searches

**Table 2:** Specific inhibition using dilutions of cell-free culture filtrates from *Pseudomonas syringae *pv.*syringae *UMAF0158 and its derived mini*Tn5 *and insertion mutants grown in liquid minimal medium (PMS).

Bacterial strains	Mangotoxin production	Dilutions of cultures filtrates^a^				
		**1:1**	**1:2**	**1:4**	**1:8**	**+ ornithine**
		
*Wild type*						
UMAF0158	+	21.7 ± 0.4	18.2 ± 0.4	13.7 ± 0.4	9.5 ± 0.5	< 7
*miniTn5 mutants*						
UMAF0158-3νH1	-	< 7	< 7	< 7	< 7	< 7
UMAF0158-6νF6	-	< 7	< 7	< 7	< 7	< 7
*pCG2-6 complementation*						
UMAF2-6-3H1	+	19.0 ± 1.0	15.5 ± 0.5	13.5 ± 0.5	9.5 ± 0.5	< 7
UMAF2-6A	+	19.0 ± 0.7	16.2 ± 0.4	12.7 ± 1.3	10.5 ± 0.5	< 7
*Insertion mutants*						
UMAF0158::ORF1	+	20.2 ± 1.3	17.0 ± 0.7	14.7 ± 0.8	11.0 ± 0.8	< 7
UMAF0158::ORF2	+	19.7 ± 1.5	16.2 ± 0.8	12.2 ± 1.1	< 7	< 7
UMAF0158::*mgo*B	+	17.7 ± 0.8	14.2 ± 0.8	12.0 ± 0.8	< 7	< 7
UMAF0158::*mgo*C	-	< 7	< 7	< 7	< 7	< 7
UMAF0158::*mgo*A	-	< 7	< 7	< 7	< 7	< 7
UMAF0158::*mgo*D	-	< 7	< 7	< 7	< 7	< 7
*pLac complementation*						
UMAF0158-6νF6 containing pLac56	+	19.2 ± 0.4	15.7 ± 0.8	12.7 ± 1.2	< 7	< 7
UMAF0158-6νF6 containing pLac6	-	< 7	< 7	< 7	< 7	< 7

The inhibition analysis was performed by *Escherichia coli *growth inhibition test

a) Toxic activity is expressed as diameter of inhibition zone (in mm). Average and standard deviation values were obtained from three replicate of three independent experiments

The four genes downstream of ORF2 exhibit a high degree of identity to four consecutive *P. syringae *pv. *syringae *B728a genes (Psyr_5009 to Psyr_5012) (Table 1). The *mgo*B gene, which contains a putative RBS at nucleotide -8 (AGGA), is 96% similar to Psyr_5009, which encodes a conserved hypothetical protein. The *mgo*B mutant UMAF0158::*mgo*B produced mangotoxin (Table 1), although the level of mangotoxin was decreased slightly (Table 2). A search of the Pfam database revealed a similarity to DUF3050, a protein of unknown function, between amino acids 15 and 244 with an e-value of 3.1e-97. Searches in the InterProScan (EMBL-EBI) database revealed that the theoretical MgoB protein product is similar to the haem oxygenase-like, multi-helical superfamily between amino acids 128 and 245 (e-value of 1.3e-8).

The inactivation of the *mgo*C, *mgo*A and *mgo*D genes yielded mutants (UMAF0158::*mgo*C, UMAF0158::*mgo*A and UMAF0158::*mgo*D) that were completely unable to produce mangotoxin (Tables 1 and 2). The *mgo*C gene, which contains a putative RBS at -7 (AAGGA), exhibits 95% similarity to the Psyr_5010 gene of *P. syringae *pv. *syringae *B728a, a conserved hypothetical protein (Table 1). Homology searches for the MgoC protein product in the Pfam database revealed a significant match with the p-aminobenzoate N-oxygenase AurF from *Streptomyces thioluteus*. The alignment was between amino acids 2 and 295 with an e-value of 7.2e-88. The disruption of *mgo*A by insertion (UMAF0158::*mgo*A) or mini*Tn5 *(UMAF0158-6γF6) mutation resulted in the complete inactivation of a putative nonribosomal peptide synthetase (NRPS). The inactivation of *mgo*A has previously been shown to result in defects in mangotoxin production and considerably reduced virulence [15]. However, a putative RBS for *mgo*A could not be located using the consensus sequences published to date. Finally, insertional mutagenesis of the *mgo*D gene, which contains a putative RBS at -6 (ATGGAG), resulted in the inactivation of a conserved hypothetical protein that is 94% identical to Psy_5012. A conserved-domain analysis of the hypothetical amino acid sequence of MgoD revealed sequence similarity to Polyketide_cyc2, a polyketide cyclase/dehydrase and lipid transporter domain, from amino acids 20 to 158. The e-values were 1e-17 (Specialized BLAST-NCBI) and 1.6e-23 (Pfam).

### The genetic organisation of the *mgo *operon and complementation of insertional mutants

**Figure 2:**
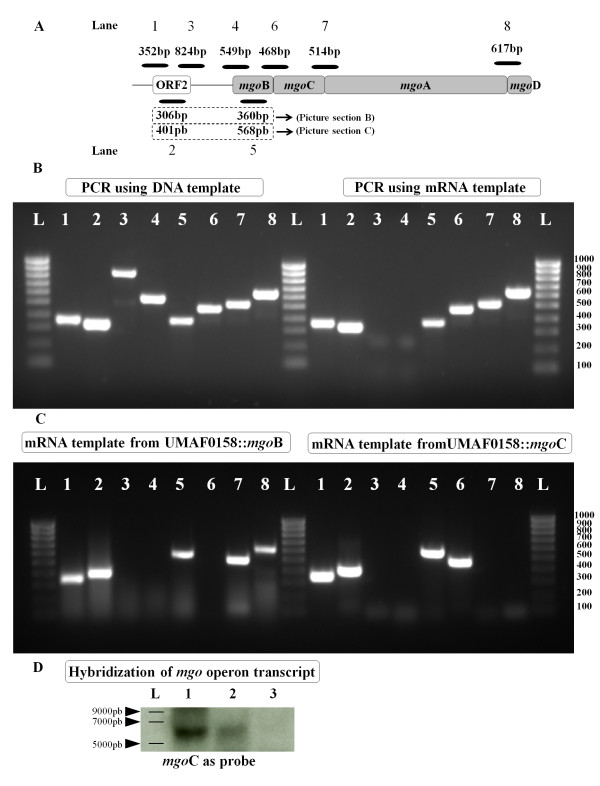
**Characterisation of the *mgo *operon**: **A**) diagram of the location of the amplified region obtained during the RT-PCR experiments. The molecular size and gel lanes are indicated. Lanes 2 and 5 have two molecular sizes: lane 2 shows 306 bp, and line 5 shows 360 bp in section B; lane 2 shows 401 bp and lane 5 shows 568 bp in section C. The putative *mgo *operon involved in mangotoxin production by *Pseudomonas syringae *pv. *syringae *UMAF0158 is illustrated by grey boxes, and the upstream ORF is indicated by a white box. Each gene studied in this study was given a specific name. **B**) The PCR products obtained from the RT-PCR experiments that used as templates genomic DNA and mRNA derived from wild-type UMAF0158 after 48 h of incubation at 22°C on liquid PMS minimal medium. **C**) The PCR products obtained from the RT-PCR experiments using mRNA from the insertional mutants UMAF0158::*mgo*B and UMAF0158::*mgo*C. HyperLadder IV (Bioline) were subjected to agarose electrophoresis. **D**) The Northern blot analysis of the total mRNA obtained from wild-type UMAF0158 and the insertional mutants using a fraction of the *mgo*C gene as a probe. Lane L, ssRNA ladder; lane 1, UMAF0158; lane 2, UMAF0158::*mgo*B and lane 3, UMAF0158::*mgo*C.

To define the *mgo *operon and determine its genetic organisation and co-transcription, reverse-transcription PCR (RT-PCR) experiments were performed (Figure 2). The total DNA and RNA from wild-type UMAF0158 grown in PMS minimal medium at 22°C were used, and the RT-PCR primers were designed to anneal between the ORFs. The total DNA was used as an amplification control, and the cDNA derived from the mRNA was used to detect the transcripts of genes belonging to the putative *mgo *operon. To confirm the co-transcription of *mgo*B, *mgo*C, *mgo*A and *mgo*D, we amplified the connecting areas between the sequential ORFs of the putative *mgo *operon (Figure 2A). Sequences within ORF2 and *mgo*B were also amplified to determine their mRNA transcripts (Figure 2A, B). Our results indicated that ORF2 and the upstream region and *mgo*B and the downstream region were amplified. However, there was no amplification of the inter-genetic region upstream of *mgo*B. These results suggest that the transcriptional unit is *mgo*B, *mgo*C, *mgo*A and *mgo*D (Figure 2B). The lack of amplification between ORF2 and *mgo*B supports the presence of a putative promoter in this DNA sequence.

Additional RT-PCR experiments showed that only the disrupted *mgo*B gene was not amplified in UMAF0158::*mgo*B while the transcripts of the disrupted *mgo*C gene as well as that of the downstream genes were absent in UMAF0158::*mgo*C (Figure 2C). A hybridisation analysis of the transcript of the *mgo *operon with the total mRNA from wild-type UMAF0158 and the insertional mutants UMAF0158::*mgo*B, and UMAF0158::*mgo*C showed that the transcript was present in the wild-type strain and reduced in the *mgo*B mutant strain (Figure 2D).

To confirm the role of these genes in mangotoxin production and to analyse the specific phenotype of each mutation, we performed a complementation analysis using plasmids containing all of the genes that were situated downstream of the mutations (Table 3). The *mgo *genes were cloned downstream of the P_LAC _promoter. Plasmid pLac36, which contains the structural genes of the operon (*mgo*B, *mgo*C, *mgo*A and *mgo*D), and a plasmid containing the genomic clone pCG2-6 were both able to restore mangotoxin production in all of the constructed mutants (Tables 3 and 2). These results demonstrate that the complemented plasmids were functional and rule out the possibility that secondary mutations influence mangotoxin production. Plasmid pLac56, which contains only *mgo*A and *mgo*D, was able to complement the phenotypes of the mini*Tn5 *mutant UMAF0158-6γF6 and the insertional mutants UMAF0158::*mgo*A and UMAF0158::*mgo*D. Plasmid pLac6, however, was only able to complement UMAF0158::*mgo*D (Table 3). These complementation experiments show that the insertional mutants UMAF0158::*mgo*C, UMAF0158::*mgo*A and UMAF0158::*mgo*D were unable to produce mangotoxin even when the downstream genes were restored on a plasmid. The insertional mutation of the *mgo*C, *mgo*A and *mgo*D genes resulted in a loss of mangotoxin activity, which did not occur when *mgo*B was mutated (Tables 1 and 2). Therefore, we cannot eliminate the possibility that a polar effect of the insertional mutations affected the phenotypes of the mutants and downstream genes transcription. Apparently the insertional mutation in *mgo*B did not show polar effect on *mgo *genes located downstream (*mgo*C, *mgo*A and *mgo*D), in contrast with the insertional mutation in *mgo*C, which produce a polar effect on *mgo *downstream genes transcription (Figure 2, Table 3).

**Table 3:** Analysis of mangotoxin production using mini*Tn5 *and insertional mutants obtained from *Pseudomonas syringae *pv.*syringae *UMAF0158, before and after transformation with plasmids containing total or partial *mgo *operon involved in mangotoxin production

		Mangotoxin production in strains containing complementing plasmids^a^				
		
Strain	ORF mutated	None	pCG2-6	pLac36	pLac56	pLac6
UMAF0158	na^b^	+	na	na	na	na
UMAF0158::*mgo*B	*mgo*B	+	+	+	+	+
UMAF0158-3νH1	*mgo*C	-	+	+	-	-
UMAF0158::*mgo*C	*mgo*C	-	+	+	-	-
UMAF0158-6νF6	*mgo*A	-	+	+	+	-
UMAF0158::*mgo*A	*mgo*A	-	+	+	+	-
UMAF0158::*mgo*D	*mgo*D	-	+	+	+	+

a) +: presence of inhibition halo; -: absence of inhibition halo

b) na, not applicable

pCG2-6: genomic clone of UMAF0158. GenBank access DQ532441 (Table 4)

pLac36: *mgo*B, *mgo*C, *mgo*A and *mgo*D cloned in pBBR1MCS-5 (Table 4)

pLac56: *mgo*A and *mgo*D cloned in pBBR1MCS-5 (Table 4)

pLac6: *mgo*D cloned in pBBR1MCS-5 (Table 4)

### Mangotoxin production in mutants derived from *Pseudomonas syringae *pv. *syringae *UMAF0158

To further support our results, we determined the amount of mangotoxin production in the insertional and mini*Tn5 *mutants relative to wild-type UMAF0158 (Table 2). The production of the syringomycin complex by the insertional mutants confirmed that only mangotoxin production was affected (data not shown). The results obtained from the quantitative mangotoxin analysis indicated that the two mini*Tn5 *mutants that were complemented with pCG2-6, UMAF2-6A and UMAF2-6-3H1, and the insertion mutant UMAF0158::ORF1 were able to produce mangotoxin at the same level as wild-type UMAF0158.

Upon complementation with pLac56 (*mgo*A and *mgo*D), mangotoxin production was restored in the mutants UMAF0158::ORF2 and UMAF0158::*mgo*B and the mini*Tn5 *mutant UMAF0158-6γF6; however, the production was slightly lower and could be detected only until a 1:4 dilution (Table 2).

### Promoter and terminator localisation in the mgo operon

Promoter expression and terminator localisation experiments were performed to characterise the structure of the operon.

**Figure 3:**
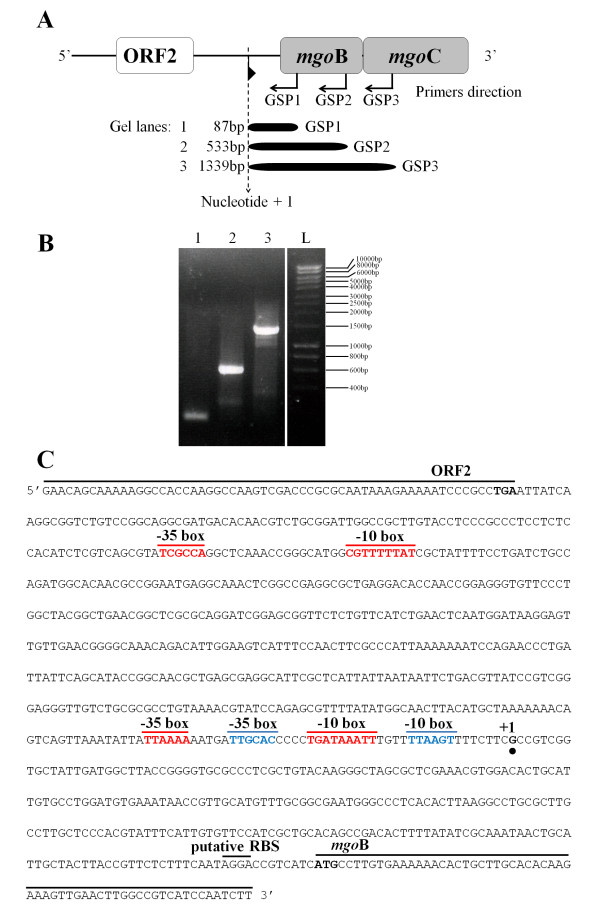
**Localisation and analysis of the promoter in the *mgo *operon**. **A**) The design of the 5' RACE experiment, including the upstream and downstream sequences of the *mgo*B gene. **B**) The results obtained from the 5' RACE experiment. Lane 1, amplification from the primer GSP1; lane 2, amplification from the primer GSP2; lane 3, amplification from the primer GSP3; lane L, loading buffer and HyperLadder I (Bioline), with the different sizes indicated. **C**) The 3'-end of ORF2, with the stop codon in bold type, and the 5'-end of *mgo*B, with the start codon also in bold type, are indicated. The nucleotide sequence (814 bp) located between these two ORFs was analysed. The two putative promoters found in this sequence by the *in silico *analysis are indicated by the locations of the respective -10 and -35 boxes (in red); moreover, the sequence of the alternative -35 and -10 boxes, which are more closely related to *Pseudomonas *promoters, are marked in blue. The start of the transcript is marked as nucleotide +1 (with black point under the nucleotide). The putative ribosomal binding site (RBS) of *mgo*B is also indicated.

**Figure 4:**
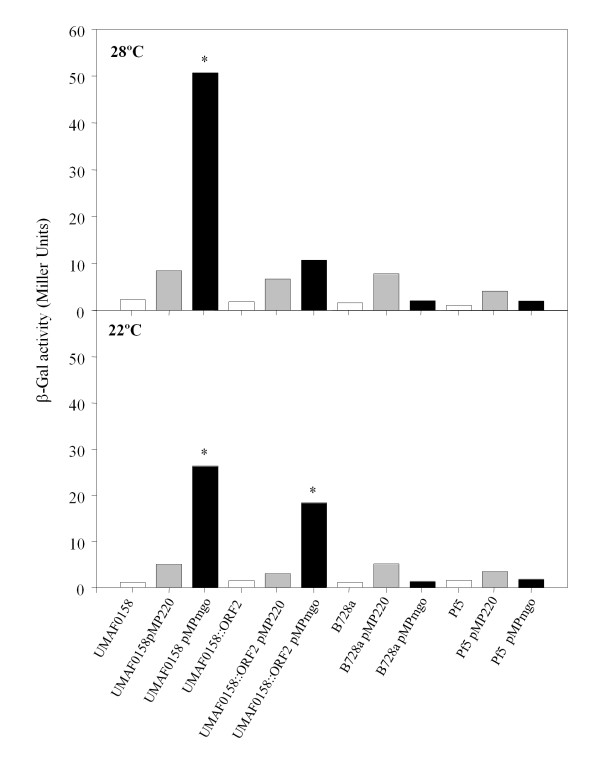
**The β-galactosidase (β-Gal) expression of *Pseudomonas syringae *pv. *syringae *wild-type UMAF0158, the UMAF0158::ORF2 insertional mutant, *Pseudomonas syringae *pv. *syringae *B728a and *Pseudomonas fluorescens *Pf5 was detected on PMS minimal medium (without manipulation (**□**), transformed with empty promoter-probe vector pMP220 (Grey Column) and transformed with pMPmgo, which contains the putative promoter P*_mgo _*(■))**. The cultures were tested at 28°C and 22°C. The results are indicative of three experiments performed in triplicate. The data were analysed by an analysis of variance (ANOVA) using SPSS 8.0 software for Windows (SPSS Inc., Chicago, IL, USA). The columns labelled with an asterisk are significantly different (*P *< 0.01) according to the least significant difference (LSD) test.

The promoter prediction software BPROM (SoftBerry Inc.) was used to identify possible promoters in the putative *mgo *operon. The best candidates were found in the nucleotide sequence (814 bp) of the non-coding region located upstream of the *mgo*B gene. Two possible promoters were predicted and designated as P*_mgo_*. The first predicted promoter was located at position 134 from 5'-end with a linear discriminant function (LDF) of 0.59, a -10 box, CGTTTTTAT, at position 119 (score: 37) and a -35 box, TCGCCA, at position 95 (score: 24). The second predicted promoter was located at position 549 from the 5'-end of the sequence, with an LDF of 4.38, a -10 box, TGATAAATT, at position 534 (score: 55) and a -35 box, TTAAAA, at position 513 (score: 37) (Figure 3C). The scores of the first predicted promoter were lower than those of the second promoter. According to the *in silica *prediction, the 814 bp sequence containing both putative promoters was cloned into pMP220, and its activity was measured with a β-galactosidase assay (β-Gal) [17,18]. The P*_mgo _*studies were performed in *Pseudomonas fluorescens *Pf-5, which contains no genomic sequences that are homologous to the *mgo *operon, and *P. syringae *pv. *syringae *B728a, which contains genomic sequences that are homologous to the *mgo *operon but cannot produce mangotoxin. We also used the insertional mutant UMAF0158::ORF2, which contains a disruption in the putative transcriptional regulator gene, and wild-type UMAF0158. P*_mgo _*activity was measured in three different culture media (LB, KB and PMS) and at two growth temperatures (28°C and 22°C). In the minimal medium PMS, the P*_mgo _*promoter was active in the wild-type strain at both temperatures and in the insertional mutant at 22°C (Figure 4). The β-Gal assays of the strains grown in rich LB and KB media did not indicate activity in any of the strains at either temperature (data not shown).

Once the presence of promoter activity in the analysed sequence was confirmed, the 5'RACE method was used to determine the transcript start point of the *mgo *operon (Figure 3A, B). With this method, we could determine which of the two putative promoters of the *mgo *operon was the functional promoter and also analyse the presence of an additional promoter between *mgo*B and *mgo*C, which was suggested by the results of the polarity and mangotoxin production experiments. The start point of the transcript (nucleotide +1) was located 18 nucleotides after the predicted -10 box of the second putative promoter (Figure 3C). Therefore, the second predicted promoter appears to be the functional promoter for the *mgo *operon. At this point using the known nucleotide sequence and the 5'RACE results, alternative -35 and -10 boxes were located in correct positions from nucleotide +1. The sequences of these alternative -35 and -10 boxes are more typical of *Pseudomonas *sigma70-dependent promoter sequences [19,20] than the predicted boxes by BPROM software, which are similar to *Escherichia coli *sequences (Figure 3C). Additionally, the results do not support the presence of an alternative promoter at the end of *mgo*B, which could explain the previous results.

**Figure 5:**
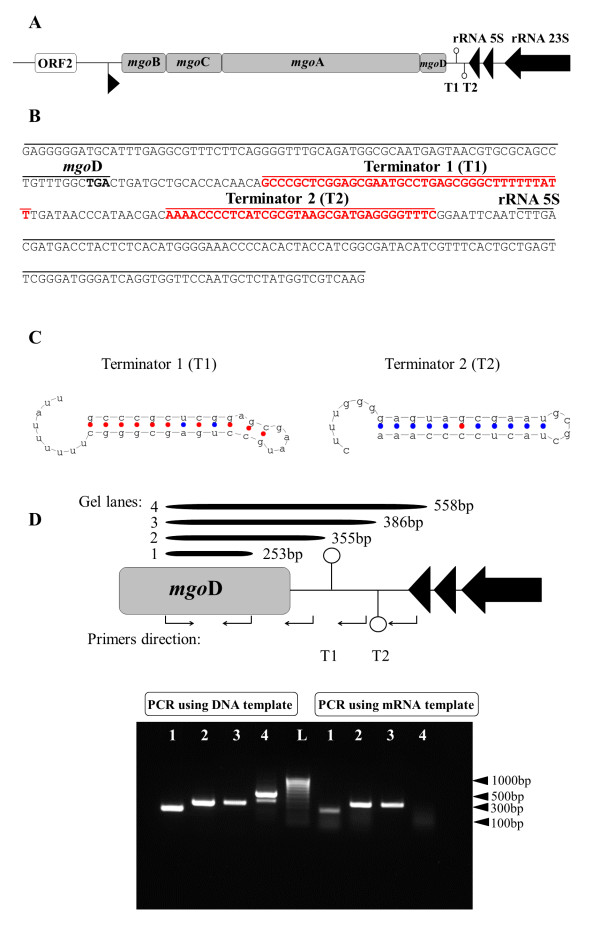
**Study of the terminators located at the end of the *mgo *operon**. **A**) The organisation of the *mgo *operon, showing the genes belonging to the operon as grey boxes, the ORF outside the operon as a white box and the rRNA as black arrows; the promoter (►) and transcriptional terminators (○) are indicated as T1 and T2. **B**) The terminal region of the *mgo *operon, the 3'-end of the *mgo*D gene (with the stop codon in bold type) and the 5'-end of the 5S rRNA are indicated. Between these two segments is the nucleotide sequence in which the two putative terminators were identified by the bioinformatic analysis (SoftBerry Inc.), which are indicated as terminator 1 (T1) and terminator 2 (T2). **C**) The secondary structure of the two putative Rho-independent terminators within the *mgo *operon (terminator 1 (T1) and terminator 2 (T2)), as predicted by FindTerm software (SoftBerry Inc.). **D**) A diagram of the experimental design for locating the functional *mgo *operon terminator. The amplicon sizes and primer directions are indicated. Agarose electrophoresis of the RT-PCR experiments. HyperLadder IV (Bioline) was used as the loading buffer.

The location of the transcriptional terminator was then determined. A 118-bp sequence was located in the region downstream of the *mgo *operon (Figure 5A) and was compared with the equivalent DNA segment in Pss B728a by Blast (NCBI). A putative terminator (CCC CTC ATC GCG TAA GCG ATG AGG GG), which was 100% identical to the equivalent terminator in Pss B728a, was identified at position 79 from the *mgo*D stop codon. This terminator sequence was then analysed by FoldRNA software (SoftBerry Inc.), a program used to predict RNA secondary structure through energy minimisation, to calculate the free energy released during palindrome structure formation. A value of -24.4 kcal/mol was found in 84% of the helices. The entire sequence of 118 bp was also analysed by FindTerm software (SoftBerry Inc.) to locate putative Rho-independent bacterial terminators. Two putative terminators (T1 and T2) were found, the first (T1) of which contained more apparent poly-U tracts typical of Rho-independent terminators (Figure 5B, C). T1 was located at position 20-57 (-12.5 kcal/mol and 35% in helices), and T2 was located at position 75-108 (-24.9 kcal/mol and 40% in helices), which includes the sequence homologous to the B728a terminator. Both terminator sequences had negative free energy values, indicating that their folding would be favoured and spontaneous. Finally, to determine which putative terminator acted as the functional terminator, RT-PCR experiments were performed by amplifying the 3'-end of the transcript with primers designed to anneal before, in the middle of and after of the putative terminators (Figure 5D). The amplification test of the *mgo *transcript revealed that the T1 sequence but not the T2 sequence was included in the *mgo *transcript, indicating that T1 is the functional terminator of the *mgo *operon.

### The hypothetical function of the *mgo *operon

Our study of the *mgo *locus demonstrates that the *mgo *operon is involved in the biosynthesis or regulation of mangotoxin. Recent studies of the *pvf *genes, which share high homology with the *mgo *operon, have indicated a possible regulatory function for those genes [21]. Given these findings, it should be possible to isolate a signalling molecule that is required for virulence gene expression and use it to restore the virulence of an *mgo*A mutant (defective in the nonribosomal peptide synthetase [15]) by adding this molecule to the growth medium. Growing the UMAF0158 mutant, which possesses a deletion of *mgo*A (UMAF0158ΔmgoA) and is defective in mangotoxin production, in media supplemented with an extract from wild-type UMAF0158 restored mangotoxin production. An extract from the *mgo*A mutant did not restore toxin production. Strains that were defective in other regulatory genes were also used. Extracts from wild-type Pss UMAF0158 and the reference strain Pss B728a were used to complement UMAF0158-2βB7, which contains a mini*Tn5 *disruption of the *gac*A gene, and UMAF0158-3αE10, which contains a mini*Tn5 *disruption of the *gac*S gene (Table 4). Mangotoxin production was restored in the defective mutants when an extract from UMAF0158 was added. By contrast, an extract from Pss B728a only restored mangotoxin production in the *gac*S mutant (Table 4). These results suggest a possible regulatory role for the *mgo *operon.

**Table 4:** Extract complementation of defective mutants in mangotoxin production using extract obtained from *Pseudomonas syringae *pv.*syringae *wild-type UMAF0158 and references train B728a

	Controls		Extracts	
	
Complemented strains	Standard	methanol	UMAF0158	B728a
*P. syringae *pv*. syringae*				
UMAF0158	+	+	nd	nd
B728a	-	-	nd	nd
*Defective mutants*				
UMAF0158ΔmgoA	-	-	+	-
UMAF0158-2βB7 (*gac*A)	-	-	+	-
UMAF0158-3αE10 (*gac*S)	-	-	+	+

## Discussion

The focus of the present study was to characterise the transcriptional organisation that is directly involved in mangotoxin production. We had previously identified the *mgo *operon (Mangotoxin-Generating Operon) [15]. We determined which genes are involved in mangotoxin production by disrupting each chromosomal gene that was previously identified in pCG2-6 by mutagenesis. The disruption of ORF0 and ORF1 did not affect mangotoxin production. These two genes may belong to another independent gene cluster located close to the *mgo *operon that is not involved in mangotoxin production. ORF2 transcription was independent of the *mgo *operon, and ORF2 is homologous to the GntR family of transcriptional regulators. This family of regulatory proteins consists of the N-terminal HTH region of GntR-like bacterial transcription factors. An effector-binding/oligomerisation domain is usually located at the C-terminus [22]. In the deposited genomes of other *P. syringae *pathovars, the genes in this family are often located close to gene clusters that are homologous to the *mgo *operon. The relationship between ORF2 and the regulation of the *mgo *operon remains unclear. In the present study, we observed promoter P*_mgo _*expression in the ORF2 mutant (UMAF0158::ORF2) when it was grown in minimal medium at 22°C but not at 28°C, in agreement with the production of mangotoxin by the ORF2 insertional mutant. These data suggest that ORF2 is not involved in mangotoxin production but provide no direct information on the possible influence of ORF2 on the *mgo *operon with respect to variations in temperature.

Our results demonstrate that the DNA sequence downstream of ORF2 constitutes an operon. Ma *et al*. [23] first established the correlation between the presence of a Shine-Dalgarno sequence, also known as a ribosomal binding site (RBS), and translational initiation, the expression levels of the predicted genes and operon structure [23]. We found putative RBSs in almost all of the genes in the putative *mgo *operon. Only the *mgo*A gene, in which the start codon overlaps with the stop codon of *mgo*C, does contain a potential RBS sequence. *mgo*C and *mgo*A may share the same RBS, and post-translational changes may separate the two proteins; this situation could explain the absence of a putative RBS for the *mgo*A gene. The mutagenesis and bioinformatics analysis of each gene in the *mgo *operon provided insight into their relationship to mangotoxin production. The disruption of *mgo*B did not abrogate mangotoxin production; however, the production decreased noticeably compared with the wild-type strain. Protein domain searches indicated that *mgo*B is similar to haem oxygenase. This enzyme is a member of a superfamily represented by a multi-helical structural domain consisting of two structural repeats that is found in both eukaryotic and prokaryotic haem oxygenases and in proteins that enhance the expression of extracellular enzymes [24]. The disruption mutants of the next three genes, *mgo*C, *mgo*A and *mgo*D, were unable to produce mangotoxin, indicating that these genes are essential for mangotoxin production. A similar conclusion was reached by Aguilera *et al*. [10], who obtained Tox^- ^phenotypes when 11 different genes in the *pht *cluster, a region involved in phaseolotoxin production, were mutated by insertion, indicating that all of the genes located within this region encode proteins that are required at different stages of phaseolotoxin production, including synthesis, transport and regulation [10].

The sequence analysis of *mgo*C prompted us to search the superfamily protein domains, revealing a similarity to the N-oxygenase domain. This domain was identified in the protein PrnD, which is derived from the pyrrolnitrin biosynthesis gene cluster of *Pseudomonas fluorescens*. MgoC is also similar to AurF from *Streptomyces thioluteus*, which produces the starter unit p-nitrobenzoic acid (PNBA) for the polyketide synthase of the aureothin biosynthesis pathway [25]. The gene *mgo*A, which is homologous to non-ribosomal peptide synthetases, is the largest gene in the *mgo *operon, and its disruption produces a mutant that is defective in mangotoxin production. Its structure, participation in mangotoxin production and influence on the virulence of the wild-type bacterium has been discussed previously [15]. The final gene studied was *mgo*D; a domain localisation analysis indicated that *mgo*D could be a Polyketide_cyc2 belonging to the star-related lipid-transfer (START) domain superfamily. The START superfamily includes bacterial polyketide cyclase/aromatases and two families of previously uncharacterised proteins that are present only in plants and the cyanobacterium *Prochlorococcus *[26].

After analysing the elements that composed the putative *mgo *operon, we evaluated whether the four genes were transcribed together in a single transcript. RT-PCR experiments using the wild-type RNA showed that the four genes were connected in the single transcript (Figure 2). Moreover, the transcript size was analysed by hybridisation, which confirmed the presence of a single transcript with a sufficient size (about 6 kb) to contain the genes *mgo*BCAD; however, the exact size of the transcript could not be determined.

Following the identification of the *mgo *operon, the promoter and transcription terminator were identified and studied. The *in silico *analysis of the sequence identified two putative promoters. Promoter activity was detected only in a minimal medium, the same culture medium that is traditionally used for antimetabolite toxin assays [2,13]. Promoter activity occurred in the wild-type strain at both temperatures and in the ORF2 insertion mutant at 22°C only. The other *Pseudomona*s *spp*. experimental strains, which do not produce mangotoxin, did not exhibit any β-Gal activity. The promoter activity in the wild-type strain was more intense at 28°C than 22°C. When the promoter activity was assayed at 22°C, the activity of the mutant UMAF0158::ORF2 was statistically comparable with that of the wild-type strain. These results suggest a possible influence of ORF2 on the *mgo *operon during its regulation in response to temperature variations. The promoter inactivity in the other two strains of *Pseudomonas spp*. may be due to the absence of genes homologous to the *mgo *operon in *P. fluorescens *Pf-5, but this explanation is not applicable to Pss B728a. The sequence in B728a that is homologous to the *mgo *operon is composed of genes that are orthologous to the *mgo *genes; theoretically, the promoter activity should have been similar to that of the wild-type strain, but it was not. This result suggests that there are additional genes that are necessary for mangotoxin production that are not present in B728a. In support of this explanation, additional genes involved in mangotoxin production have been identified in UMAF0158 and cloned into a different vector than pCG2-6 [15]. The initial sequence analysis did not show any identity with the genome of B728a, and thus these additional genes may influence *mgo *promoter activity.

Finally, the functional promoter of the *mgo *operon was established by locating the start of the *mgo *transcript (Figure 4), which is located 18 nucleotides after the putative -10 box of the second promoter analysed *in silico*. Thus, the first putative promoter was eliminated as a functional promoter of the *mgo *operon. Once the +1 site was established, it was possible to locate additional -35 and -10 boxes, which were typical of sigma70 dependent promoters of *Pseudomonas spp *[19,20] and were more closely related than the predicted -35 and -10 boxes by BPROM software developed for *Escherichia coli*, which are less accurate in the search for promoters of *Pseudomonas spp*. These results allowed us to determine the functional promoter of the *mgo *operon. The *mgo *operon terminator was found in a similar manner. The *in silico *analysis of the sequence identified two possible terminator sequences between the 3'-end of *mgo*D and the 5'-end of the 5S rRNA, both of which exhibited secondary structures typical of transcription terminators. We considered that the ribosomal transcript terminator is also likely present in the analysed sequence. RT-PCR was used to clarify which was the operon terminator, establishing T1 as the functional terminator of the *mgo *operon. This is a typical terminator with a stable hairpin having many GC pairs followed by a string of T's. So, it seems that the T1 terminator is a bifunctional terminator, serving this DNA region to terminate transcription of *mgo *operon in the sense strand and of the ribosomal operon in the antisense strand (Figure 5).

The results described above are sufficient to suggest that *mgo*BCAD is a transcriptional unit and therefore propose that *mgo *is an operon. If this argument is correct, mutations in each *mgo *gene should lead to the absence of a transcript for the downstream genes. A polar effect was demonstrated for UMAF0158::*mgo*C but not UMAF0158::*mgo*B. The mutation in *mgo*B did not prevent the transcription of the downstream genes, although the hybridisation experiments revealed that the transcription appeared to be less efficient. This reduction in transcription corresponds to the reduced production of mangotoxin by UMAF0158::*mgo*B relative to the wild-type strain. Therefore, the results obtained with wild-type UMAF0158 and the insertional mutants of *mgo*C, *mgo*A and *mgo*D support the hypothesis that the *mgo *genes form an operon in contrast, the results with the mutant UMAF0158::*mgo*B do not. We also evaluated the possible existence of an alternative promoter after the *mgo*B gene, which would explain the production of mangotoxin by the mutant UMAF0158::*mgo*B. However, during 5'RACE experiment (Figure 3) only a single transcription start site was located, eliminating the possibility of another promoter downstream of *mgo*B. Therefore there must be something different between the mutant and wild-type strain, which is probably the plasmid integration. In reviewing the process by which the *mgo *mutants were obtained, we observed that UMAF0158::*mgo*B was not easy to obtain. The size of *mgo*B is 777 bp, and the cloned sequence in pCR2.1 was 360 bp of *mgo*B. The integration of pCR::*mgo*B into *mgo*B occurred by single-crossover homologous recombination as it was confirmed. During this process, the plasmid could be integrated into *mgo*B sequence maintaining an important part of the gene. In this circumstances *mgo*B or sufficient fragment of it, and the remarkably other three genes of the *mgo *operon, could be under the influence of a promoter located in plasmid polylinker, *lac*Z promoter, allowing a reduced transcript expression (Figure 2) and mangotoxin production (Tables 1 and 2). To determine the insert position, a PCR was performed in which the forward primer annealed to the *lac*Z gene (M13F primer) and the reverse primer annealed to the 5'-end of the *mgo*C gene, with wild-type UMAF0158 used as the negative control. The amplicon obtained from the mutant UMAF0158::*mgo*B had a size of 1000 bp, confirming that the plasmid pCR::*mgo*B was integrated and the *lac*Z promoter is close to *mgo*B fragment (Additional file 1: Figure S1).

Because the chemical structure of mangotoxin is unknown [13], it is difficult to establish a hypothesis concerning the role of the *mgo *genes in mangotoxin biosynthesis or to determine whether they are related to the regulation of mangotoxin production. Recent studies in *P. entomophila *have focussed on the *pvf *gene cluster, which is homologous to the *mgo *operon, and suggest that the gene cluster serves as a regulator of certain virulence factors in pathogenic strains of *Pseudomonas spp*. The *pvf *gene cluster may be a new regulatory system that is specific to certain *Pseudomonas *species [21]. In the present study, extract complementation restored mangotoxin production in the UMAF0158ΔmgoA mutant only when the culture medium was supplemented with an extract from wild-type UMAF0158. Polar effects of the deleted *mgo*A on *mgo*D expression were excluded because the construction of the deletion mutant preserved the reading phase of protein translation. Mangotoxin production was restored in the mini*Tn5 *mutants, which contain disrupted regulatory genes, when their cultures were complemented with a wild-type extract. These results are in agreement with the results obtained by Vallet-Gely *et al*. [21], in which *pvf *and *gac *mutants were complemented by a wild-type extract. These results allow us to propose a putative regulatory role for the *mgo *operon in secondary metabolite production by *P. syringae *pv. *syringae*, in accordance with Vallet-Gely *et al*. [21].

To fully characterise the functions of the *mgo *operon, more data concerning the chemical structure of mangotoxin and a characterisation of the other genetic traits that regulate mangotoxin biosynthesis by *P. syringae *pv. *syringae *UMAF0158 are required.

## Conclusions

In the present study, the organisation of the *mgo *operon in *P. syringae *pv. *syringae *UMAF0158 was characterised. The *mgo *operon is composed of four genes, *mgo*B, *mgo*C, *mgo*A and *mgo*D. Additionally, this operon possesses one active promoter and a terminator. The last three genes are essential for mangotoxin production, as insertional mutation of these genes results in a loss of mangotoxin production. This operon is only active in minimal medium, in agreement with the standard process for mangotoxin production. Moreover, experiments performed to determine the functional role of the *mgo *operon demonstrated a putative regulatory function in the production of mangotoxin.

## Methods

### Bacterial strains and plasmids used in this study

The strains of *Escherichia coli*, *Pseudomonas fluorescens *Pf-5 and *Pseudomonas syringae *pv. *syringae *as well as the vectors and plasmids used in this study are listed in Table 5. *E. coli *was grown in Luria-Bertani medium (LB) at 37°C for 24 h. The *Pseudomonas *strains were grown routinely in King's medium B (KB) at 28°C for 48 h. Derivative mutants of *P. syringae *pv. *syringae *UMAF0158 (Table 5) were grown and maintained in KB supplemented with the appropriate antibiotics (ampicillin, 50 μg/ml; streptomycin, 50 μg/ml; kanamycin, 50 μg/ml; and gentamicin, 20 μg/ml).

**Table 5:** Bacterial strains and plasmids used in this study

Strain or plasmid	Relevant characteristics^a^	Reference or source
*Escherichia coli*		
DH5α	*rec*A *lac*ZΔM15	[27]
CECT831	Indicator strain of mangotoxin production	CECT^b^
*Pseudomonas fluorescens*		
Pf-5	Complete genome sequenced and free access.	[28]
*Pseudomonas syringae *pv. *syringae *		
B728a	Complete genome sequenced and free access	[16]
UMAF0158	Wild type isolated from mango, mangotoxin producer, Nf^r^	[12]
UMAF0158-2βB7 UMAF0158-3αE10	mini*Tn5 *mutants of UMAF0158 in *gac*A and *gac*S respectively, defective in mangotoxin, Km^r^, Nf^r^	[15]
UMAF0158-3νH1 UMAF0158-6νF6	mini*Tn5 *mutants of UMAF0158, defective in mangotoxin production, Km^r^, Nf^r^	[15]
UMAF2-6-3H1 UMAF2-6A	mini*Tn5 *mutants from UMAF0158 and complemented with plasmid pCG2-6, production of mangotoxin restored, Km^r^, Amp^r^, Nf^r^	This study
		[15]
UMAF0158::ORF0^c^	ORF0 mutant of UMAF0158, ORF0::pCR-ORF0, Km^r^, Nf^r^	This study
UMAF0158::ORF1	ORF1 mutant of UMAF0158, ORF1::pCR::ORF1, Km^r^, Nf^r^	This study
UMAF0158::ORF2	ORF2 mutant of UMAF0158, ORF2::pCR::ORF2, Km^r^, Nf^r^	This study
UMAF0158::*mgo*B	*mgo*B (ORF3) mutant of UMAF0158, *mgo*B::pCR::mgoB; Km^r^, Nf^r^	This study
UMAF0158::*mgo*C	*mgo*C (ORF4) mutant of UMAF0158, *mgo*C::pCR::mgoC; Km^r^, Nf^r^	This study
UMAF0158::*mgo*A	*mgo*A (ORF5) mutant of UMAF0158, *mgo*A::pCR::mgoA; Km^r^, Nf^r^	This study
UMAF0158::*mgo*D	*mgo*D (ORF6) mutant of UMAF0158, *mgo*D::pCR::mgoD; Km^r^, Nf^r^	This study
UMAF0158Δ*mgo*A	*mgo*A (ORF5) mutant of UMAF0158 by deletion, Nf^r^	This study
Plasmids		
pGEM-T	Cloning vector, Amp^r^	Invitrogen, California, USA
pCR2.1	Cloning vector *lac*Z, Km^r^, Amp^r^,	Invitrogen, California, USA
pBBR1MCS-5	Cloning vector, Gm^r^	[29]
pMP220	Promoter-probe vector containing a promoterless *lacZ *gene	[30]
pCR::ORF0	integrative plasmid pCR2.1 carrying ORF0	This study
pCR::ORF1	integrative plasmid pCR2.1 carrying ORF1	This study
pCR::ORF2	integrative plasmid pCR2.1 carrying ORF2	This study
pCR::*mgo*B	integrative plasmid pCR2.1 carrying *mgo*B	This study
pCR::*mgo*C	integrative plasmid pCR2.1 carrying *mgo*C	This study
pCR::*mgo*A	integrative plasmid pCR2.1 carrying *mgo*A	This study
pCR::*mgo*D	integrative plasmid pCR2.1 carrying *mgo*D	This study
pCG2-6	genomic clone of UMAF0158 GenBank-DQ532441	[15]
pLac36	From *mgo*B to *mgo*D cloned in pBBR1MCS-5	This study
pLac56	*mgo*A and *mgo*D cloned in pBBR1MCS-5	This study
pLac6	*mgo*D cloned in pBBR1MCS-5	This study
pMPmgo	pMP220 vector containing the putative promoters of *mgo *operon	This study
pEMG	integrative plasmid for deletion mutagenesis, Km^r^.	[31]
pSW-2	plasmid carrying I-SceI gene for deletion mutagenesis, Gm^r^.	[31]

a) Amp^r^: ampicillin resistance; Gm^r^: gentamycin resistance; Km^r^: kanamycin resistance; Nf^r^: Nitrofurantoin resistance. b) CECT: Spanish Type Culture Collection. c) ORF0 was named in this way because it was cloned as an uncompleted ORF

### Detection of *P. syringae *toxin production

Syringomycin complex production by strains of *P. syringae *strains was detected using growth inhibition tests performed on potato dextrose agar (PDA) against *Geotrichum candidum *[32] and nutrient agar against *Rhodotorula pilimanae *[33].

Mangotoxin production was assayed using the indicator technique, which has been described previously and involves growth inhibition of *Escherichia coli *on Pseudomonas Minimal Medium (PMS [34]). Briefly, a double layer of the indicator microorganism was made with *E. coli *CECT831. After solidification, the *P. syringae *wild-type strain and its derivatives mutants were stabbed, and the plates were incubated at 22°C for 24 h, followed by an additional 24 h at 37°C. To determine the identity of the biochemical step that is putatively targeted by mangotoxin, the same plate bioassay was performed in separate plates with the addition of 100 μl of a 6 mM solution of ornithine or N-acetyl-ornithine. To assess the production of mangotoxin in liquid cultures, we used a cell-free filtrate dilution as previously described [13].

### Insertional inactivation mutagenesis

Insertional inactivation mutagenesis of *P. syringae *pv. *syringae *UMAF0158 was used to suppress the production of mangotoxin by inserting disruption vectors into the different ORFs of the *mgo *operon by single-crossover homologous recombination. To construct the integrative plasmids (Table 4), DNA fragments of the different ORFs within the gene cluster were obtained by PCR using primers specific to the sequence of the genomic clone pCG2-6 (accession number DQ532441) [15]. PCR, cloning and plasmid purification were carried out following standard procedures. The plasmids were transformed into the wild-type strain UMAF0158 by standard electroporation. The mangotoxin-deficient phenotype of the mutants was evaluated by the mangotoxin production assay described previously. Additionally, the mutants were analysed by PCR and Southern blot analyses using the antibiotic resistance cassette or partial target gene sequences as probes to confirm gene disruption and select single-copy transformants.

### Complementation experiments

To prevent potential polar effects from the mutations introduced into the *mgo *operon, a series of plasmids containing the mutated ORF in addition to each of the downstream ORFs located within the operon was constructed. To ensure expression, these constructs were fused to the P_LAC _promoter, which is constitutively activated in *P. syringae*. A fragment containing *mgo*B, *mgo*C, *mgo*A and *mgo*D (7808 bp) was amplified by PCR from UMAF0158 using the primers ORF3F (5'- CTG CAC AGC CGA CAC TTT TA -3') and ORF6R (5'- TCC GAG GAT CCT GTT GTG GTG CAG CAT CAG TC -3'). A fragment containing *mgo*A and *mgo*D (4107 bp) was amplified from *P. syringae *pv. *syringae *UMAF0158 using the primers ORF5F (5'- CCG CCG GAT CCC ACT GGT GGC TAA CAT CGT G -3') and ORF6R; both primers contained an artificial *BamH*I site at the 5' end to facilitate cloning. The amplifications were performed with a high-fidelity Taq polymerase (Expand High Fidelity PCR System, Roche, Basel, Switzerland), and the PCR products were cloned into the vector pGEM-T (Invitrogen, California, USA). The cloned amplicons were removed from the vector by digestion with *BamH*I and individually cloned into the *BamH*I site located within pBBR1MCS-5 [29]. The amplicons were cloned in the direction of transcription downstream from the P_LAC _promoter, resulting in plasmids pLac36 (*mgo*B, *mgo*C, *mgo*A and *mgo*D) and pLac56 (*mgo*A and *mgo*D), which contained the 7.8-kb and the 4.1-kb amplicons, respectively. To obtain *mgo*D alone, pLac56 was digested with *Sal*I, and the 0.8-kb fragment containing *mgo*D was recovered and cloned into pBBR1MCS-5, resulting in pLac6. The complementing plasmids were introduced into *P. syringae *by standard electroporation.

### Preparation of RNA for RT-PCR and northern blot experiments

Pure cultures of the wild-type strain of *P. syringae *pv. *syringae *UMAF0158 were grown for 48 h at 28°C in KMB agar to prepare a bacterial suspension in PMS minimal medium that possessed an optical density of 1.0 at 600 nm (approximately 10^9 ^cfu/ml). One millilitre of this suspension was used to inoculate 50 ml of PMS minimal medium. The culture was incubated at 22°C for 48 h with orbital shaking. RNA was isolated from the bacterial culture with a commercial NucleoSpin RNA Plant kit (Macherey-Nagel GmbH & Co. KG, Germany). The RNA concentration was determined using a Nanodrop ND-1000 (NanoDrop Technologies Wilmington, DE) and was optimised up to 50 ng/μl for RT-PCR assays and 1 μg/μl for Northern blotting. The integrity of the RNA sample was assessed by agarose gel electrophoresis. RT-PCR was performed using 100 ng of RNA at a final volume of 50 μl using the Titan OneTube RT-PCR system, according to the manufacturer's instructions (Roche Diagnostics). The primers were designed by using sequences located between each gene (Additional file 2: Table S1). A 40-cycle amplification programme (94°C for 30 s, 58°C for 1 min, and 68°C for 1 min) was performed followed by a final extension cycle at 68°C for 7 min. Positive control reactions that contained DNA isolated from each corresponding bacterial strain were included in all assays.

Northern blotting was performed using a denaturing agarose gel (0.7%) and formaldehyde (2.2 M). The samples were prepared with 20 μg of total RNA in MOPS running buffer with 2.2. M formaldehyde and 50% formamide and denatured at 65°C for 10 min. The agarose gel was run for 90 min at 60 V. The RNA was transferred to a nylon membrane by capillary diffusion using 10× saline-sodium citrate buffer (SSC) and was immobilised by UV cross-linking. The hybridisation was performed with radioactively labelled probes (dCTP^32^).

### Characterisation of the mgo operon promoter

We used pMP220 [30] as the promoter-probe vector to measure transcriptional activity by β-galactosidase (β-Gal) expression. The amplicon (1008 bp), which included the putative promoter region upstream of *mgo*B, was cloned into the multicloning site using the *EcoR*I and *Pst*I restriction sites, which were not present in the cloned sequence. The resulting plasmid, pMPmgo, was transformed into multiple bacterial species (Table 5), and β-Gal assays were performed [17,18]. The protocol followed the assay described by J.H. Miller [18], except for the addition of an extra step. In our assay, the cells were pelleted and then resuspended in assay buffer to eliminate any error in the detection of β-galactosidase enzyme activity due to the effects of different carbon sources present in the growth medium.

Additionally, 5'-RACE (Rapid Amplification of cDNA Ends) experiments were performed to locate the +1 nucleotide in the *mgo *operon transcript and determine which putative promoter is active during *mgo *operon transcription. The commercial SMART™ RACE cDNA Amplification Kit (Conthech Laboratory, Inc.) was used. Moreover, mRNA from UMAF0158 was obtained by a commercial NucleoSpin RNA Plant kit (Macherey-Nagel GmbH & Co. KG), as described above.

### Extract complementation

Extracts from wild-type UMAF0158 and the mutant UMAF0158ΔmgoA were used in the complementation experiments. UMAF0158ΔmgoA, which lacks the *mgo*A gene, was created by deletion mutagenesis as described by Martinez-García and de Lorenzo [31], taking special care to maintain the reading phase downstream of the *mgo*D gene. Extractions from the culture supernatant were performed as described by Vallet-Gely *et al*. [21]. Briefly, 200 ml of bacterial culture in PMS minimal medium was pelleted by centrifugation after 7 days of growth. The supernatants were passed through a 0.2-μm filter (Millipore Corporation, Bedford, MA); the pH was adjusted to 5.0 with HCl or NaOH, and the preparation was extracted three times with dichloromethane. Initially, the preparations were extracted with 100 ml of solvent, then again with 70 ml of solvent and finally with 50 ml of solvent. The extracts were pooled, dried with anhydrous Na_2_SO_4_, filtered through Whatman paper, evaporated to dryness and dissolved in 1 ml of methanol. To supplement the growth medium with extract, 150 μl of methanolic extract was added to a 15-ml PMS culture, which was subsequently allowed to grow for 24 h. The mangotoxin production was analysed as previously described, and cell-free filtrates of UMAF0158 and UMAF0158ΔmgoA supplemented with extracts from UMAF0158 and UMAF0158ΔmgoA were tested. Cell-free filtrates from *P. syringae *pv. *syringae *UMAF0158 and UMAF0158ΔmgoA grown in PMS supplemented with 150 μl of methanol were used as controls, as were cell-free filtrates of UMAF0158 and UMAF0158ΔmgoA that were grown in PMS under standard conditions.

### Bioinformatics

Database searches were performed using the website of the National Center for Biotechnology Information (NCBI) (http://www.ncbi.nlm.nih.gov). Homology searches and the analysis of conserved protein domains were performed using the NCBI Specialized BLAST programme, the protein tools (InterProScan) of the EMBL European Bioinformatics Institute (http://www.ebi.ac.uk) and the Pfam database (http://pfam.sanger.ac.uk). The restriction maps were constructed and analysed using the JustBio website (http://www.justbio.com). The primers were designed using Primer3 online software (http://primer3.sourceforge.net). The annotation and general manipulation of sequences was performed using Artemis software (Sanger Institute, Cambridge, U.K.). The plasmid maps were constructed using the programme Plasmid Map Enhancer 3.1 (Scientific & Educational Software). The promoter prediction was performed by SoftBerry online software http://linux1.softberry.com/berry.phtml.

## Authors' contributions

EA performed the RT-PCR assays, the promoter and terminator characterisations, the mutation experiments and the complementation experiments. EA also performed the mangotoxin test, the evaluation of mangotoxin production using the insertional, deletion and mini*Tn5 *mutants and the Northern blot experiments. JM and EA designed the plasmids and created the constructs used for the complementation experiments. EA also drafted the manuscript. VJC performed the 5'-RACE experiments and the identification of the RBS sites and contributed to the mRNA extraction. FMC and AdV were responsible for initiating this study and participated in its design and coordination and the manuscript preparation. JM conceived the mutation strategy and participated in preparing the final manuscript. APG participated in helpful discussions and the creation of the final manuscript. All authors read and approved the final manuscript.

## Supplementary Material

Additional file 1 **Figure S1**. Analysis of the plasmid integration in UMAF0158::*mgo*B. The PCR was performed using the M13F primer located in the *lac*Z gene of the pCR2.1 cloning vector and the ORF4204R primer located in the 5'-end of *mgo*C. Lane L: HyperLadder I (Bioline), lane 2: UMAF0158::*mgo*B, lane 3: UMAF0158, lane 4: negative control of the PCR reaction. Click here for file

Additional file 2 **Table S1**. The annealing position and the sequence of the utilized primers in RT-PCR experiments. Click here for file
