# Supplementary figures and images for: Characterisation of the mgo operon in Pseudomonas syringae pv. syringae UMAF0158 that is required for mangotoxin production
Source: BMC Microbiol. 2012 Jan 17;12:10. doi: 10.1186/1471-2180-12-10 (PMC3298696; doi:10.1186/1471-2180-12-10)

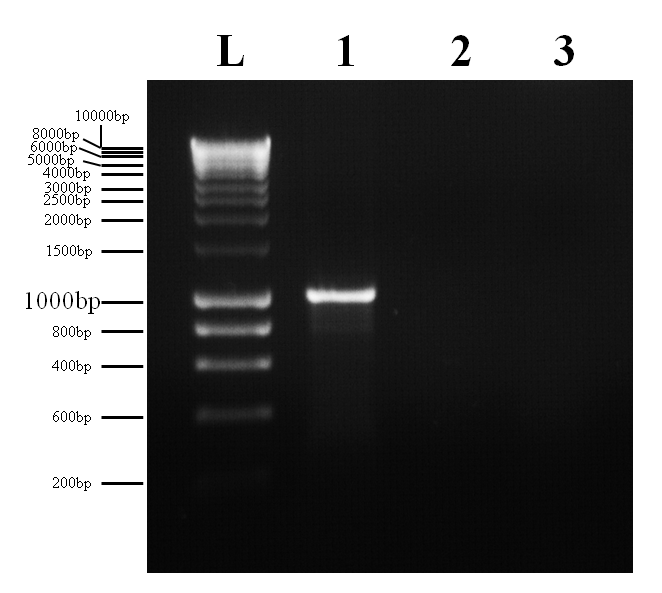

Supplement: Additional file 1 — Figure S1. Analysis of the plasmid integration in UMAF0158::mgoB. The PCR was performed using the M13F primer located in the lacZ gene of the pCR2.1 cloning vector and the ORF4204R primer located in the 5'-end of mgoC. Lane L: HyperLadder I (Bioline), lane 2: UMAF0158::mgoB, lane 3: UMAF0158, lane 4: negative control of the PCR reaction. [file 1471-2180-12-10-S1.TIFF]
