# Supplementary material for: Characterisation of the mgo operon in Pseudomonas syringae pv. syringae UMAF0158 that is required for mangotoxin production
Source: BMC Microbiol. 2012 Jan 17;12:10. doi: 10.1186/1471-2180-12-10 (PMC3298696; doi:10.1186/1471-2180-12-10)
Supplement: Additional file 2 — Table S1. The annealing position and the sequence of the utilized primers in RT-PCR experiments. [file 1471-2180-12-10-S2.PDF]

Table S1: The annealing position and the sequence of the utilized primers in RT-PCR experiments

Primer	Primer Localization in pGC2-6			Sequence
	Gene	5’ Position	3’ Position	
Pr2F	ORF2 upstream	1634	1654	CCGATGAGCGCTAAGATAGG
ORF2168R	ORF2	1967	1984	AAACAGCGTCGCCAGTTC
ORF2104L	ORF2	1921	1940	CCATTATCGATGGCAGCTTG
ORF2504R	ORF2	2301	2320	CAGGCCTTCTTCAACCAGAC
ORF2473F	ORF2	2271	2290	TATGCCAAGACCCACAACAA
Pr3R	*mgo*B upstream	3075	3094	TTATCAGGGGGTGCAATCAT
Pr3F	*mgo*B upstream	3075	3094	ATGATTGCACCCCCTGATAA
ORF3235R	*mgo*B	3604	3623	TTATCCGATTCCTCACCCAG
ORF3127L	*mgo*B	3496	3515	GATTTCATGTCGCTGACCAA
ORF3694R	*mgo*B	4044	4063	TGGCGGTCTGATAAACCTCT
ORF3594F	*mgo*B	3944	3963	ACTACCTGGAACGCCACATC
ORF4204R	*mgo*C	4392	4411	CGAAGATCACCTCATCGTTG
ORF4647F	*mgo*C	4816	4835	CTGGTACGCATCTACTGGCA
ORF5173R	*mgo*A	5305	4324	CGCACTCAGTTGTTCCTTTG
ORF53116F	*mgo*A	8248	8267	AGCCAGAACATGCGGTATTC
ORF6253R	*mgo*D	8845	8864	CGTCCCTGGAGTTCAGTTGT
ORF6141L	*mgo*D	8752	8771	ATTCATACCGGCACTGGAGA
Term1R	*mgo*D downstream	9088	9106	GTGGTGCAGCATCAGTCAG
Term2R	*mgo*D downstream	9120	9137	CCGCTCAGGCATTCGCTC
Term3R	*mgo*D downstream	9292	9309	GCATTGGAACCACCTGAT
